# Emergence of population heterogeneity in *Klebsiella pneumoniae* with a *bla*_OXA-232_-harboring plasmid: carbapenem resistance, virulence, and fitness

**DOI:** 10.1186/s12929-024-01108-4

**Published:** 2025-02-15

**Authors:** Yun Young Cho, Sun Ju Kim, Kwan Soo Ko

**Affiliations:** 1 Department of Microbiology, Sungkyunkwan University School of Medicine, Suwon, 16419 Republic of Korea; 2 Division of Infectious Diseases, Boston Children’s Hospital, Harvard Medical School, Boston, MA USA

**Keywords:** Heterogeneity, ColE-type plasmid, Carbapenem resistance, *Klebsiella pneumoniae*, *ompK36*

## Abstract

**Background:**

This study aimed to investigate the population heterogeneity on carbapenem susceptibility in *Klebsiella pneumoniae* strains that acquired a *bla*_OXA-232_-bearing ColE-type plasmid.

**Methods:**

A *bla*_OXA-232_-bearing plasmid was electroporated into two carbapenem-susceptible *K. pneumoniae* strains. High- and low-carbapenem-resistant subpopulations were identified and isolated using patch plating. The strains were subsequently subcultured in antibiotic-free media, yielding two distinct populations: a stable, high-level carbapenem-resistant strains and a heterogeneous strains. Antibiotic susceptibility tests, time-killing assays, and population profiles were conducted, along with a competition assay was performed and the growth curve analysis. To assess virulence, we performed human serum resistance and *Galleria mellonella* infection assays, and measured the expression of virulence genes using qRT-PCR. Additionally, whole genome sequencing was carried out for further anaysis.

**Results:**

Introduction of pOXA-232 into carbapenem-susceptible *K. pneumoniae* strains resulted in two isogenic transformants with distinct resistance profiles: an unstable, high-level carbapenem-resistant (HCR), and highly virulent subpopulation; and a stable, low-level carbapenem-resistant (LCR), and low-virulence subpopulation. Whole genome and expression analyses revealed dysfunctionality of *ompK36* in HCR subpopulations. Subculturing of HCR led to the re-emergence of heterogeneous populations with variations in carbapenem resistance and an additional compensatory mutation of 9,000 bp deletion in the genome. Thus, stable HCR strains featuring both mutations in *ompK36* and compensatory mutations developed.

**Conclusion:**

This study demonstrated that underlying heterogeneity can promote the emergence of stable, high-level antibiotic resistance, even with the introduction of a plasmid carrying a low-level antibiotic resistance gene, such as *bla*_OXA-232._ This highlights the critical need to closely monitor bacterial population dynamics.

**Supplementary Information:**

The online version contains supplementary material available at 10.1186/s12929-024-01108-4.

## Introduction

The emergence and spread of antibiotic-resistant strains significantly limit treatment options, with increasing resistance to carbapenems being a particularly serious concern due to its association with higher morbidity and mortality rates [1–3]. *Klebsiella pneumoniae*, a major pathogen associated with carbapenem resistance, develops resistance through various mechanisms, including upregulation of efflux pumps, reduced permeability, and the production of carbapenemases [4–8].

In addition to the traditional antibiotic resistance mechanisms, treatment is further complicated by population heterogeneity. Population heterogeneity refers to the presence of variant individuals within an isogenic population that exhibit different characteristics including variations in antibiotic susceptibility [9]. This diversity arises not only from phenotypic plasticity but also from genetic variation, and is thought to emerge as a 'bet-hedging' strategy, enhancing the survival of bacterial DNA under fluctuating environmental conditions, such as antibiotic exposure [10]. The presence of heterogeneity within bacterial populations complicates treatment because standard antimicrobial susceptibility tests, which are typically designed for homogeneous samples, may fail to detect minor subpopulations [11].

One of the most widely known population heterogeneities in bacteria is antibiotic heteroresistance, which is defined as the presence of antibiotic-resistant subpopulations within a predominantly susceptible population [12, 13]. Antibiotic-resistant subpopulations often exist at low frequencies (10^–8^ to 10^–6^), making them difficult to detect using conventional diagnostic methods [11]. However, upon exposure to antibiotics, resistant cells proliferate rapidly, potentially leading to treatment failure [14, 15]. Heteroresistance is typically transient, with a resistant subpopulation potentially reverting to susceptibility once the selective pressure of an antibiotic is eliminated [9]. However, the term heteroresistance does not fully highlight the existence of subpopulations with varying levels of antibiotic resistance within the main population [12].

These forms of heterogeneity not only aid in the overall survival of the bacterial population but also contribute to the accumulation of antibiotic resistance [12, 16]. However, the processes governing the formation, maintenance, and contribution of heterogeneity to increased antibiotic resistance remain poorly understood [17–19]. In this study, we investigated the emergence of population heterogeneity in carbapenem-susceptible *K. pneumoniae* following carbapenemase gene-borne plasmid acquisition, and its progression into a stable carbapenem-resistant phenotype. We also explored the genetic factors that contribute to population heterogeneity and development of stable carbapenem resistance.

## Materials and methods

### Bacteria and plasmid

A plasmid carrying the carbapenemase gene *bla*_OXA-232_ was obtained from *K. pneumoniae* clinical isolate M5, belonging to ST11 [20]. The ColE-type plasmid pOXA-232 is 6,141 bp in length, contains a replicase gene, *bla*_OXA-232_, a hypothetical gene, a mobile gene cassette (MOB module), a noncoding partial EreA gene associated with erythromycin resistance, and a partial *vbhA* gene. No other virulence or resistance genes were detected in strain pOXA-232. The plasmid was isolated via conjugation into DH5α for purification.

The extracted plasmid was electroporated into two carbapenem-susceptible *K. pneumoniae* ST11 strains, KCS20 and KCS22, obtained from the blood of patients. Both stains were not resistant to carbapenems with no carbapenemase genes, and demonstrated few MIC differences among each other, as shown in Table 1. KCS20 did not carry any additional plasmids, while KCS22 contained an additional plasmid of the FIIK type.

**Table 1:** Carbapenem MIC profile of parents (KCS20 and KCS22) and two of their isogenic pOXA-232 transformants (KCS20T/H and KCS20T/L, KCS22T/H, and KCS22T/L)

Antimicrobial agents	Minimum inhibitory concentration (mg/L)					
	Wild-type	Isogenic transformants		Wild-type	Isogenic transformants	
	KCS20	KCS20T/H	KCS20T/L	KCS22	KCS22T/H	KCS22T/L
Imipenem	0.5	> 64	4	0.06	> 64	4
Meropenem	0.125	> 64	4	0.03	> 64	4
Ciprofloxacin	> 64	> 64	> 64	64	64	64
Tetracycline	0.5	0.5–1	0.5	2	2	2
Amikacin	> 64	> 64	> 64	4	4	4
Colistin	0.5–1	0.5	0.5	2	2	2
Azidothymidine	> 64	> 64	> 64	> 64	> 64	> 64
Chloramphenicol	> 64	> 64	> 64	8	8	8

**Fig. 1:**
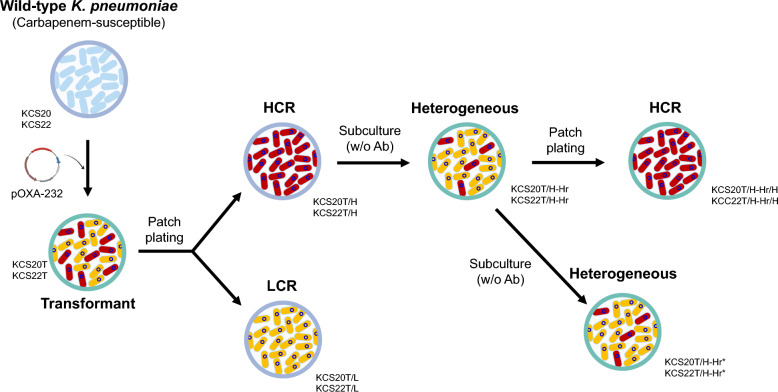
Graphical representation of experimental scheme

Electroporation was performed as described by Lee et al. [21], with minor modifications. Although electrotransformation method to transfer of the plamsid in *K. pneumoniae* isolates would not be common in nature, which may be limitation of this study, the pOXA-232 plasmid is commonly found in nature and we selected the method to investigate the effect of introduction of the plasmid. Two initial transformants, KCS20T and KCS22T were generated (Fig. 1; Supplementary Table S1). Within the two transformants, two isogenic populations exhibiting high carbapenem-resistance (HCR) phenotype (KCS20T/H and KCS22T/H) and low carbapenem-resistance (LCR) phenotype (KCS20T/L and KCS22T/L) were identified and isolated. Each population with high carbapenem resistance (KCS20T/H and KCS22T/H) was passaged daily in Luria–Bertani (LB) broth without antibiotics for 20 days, and subsequent strains with heterogeneous phenotypes (KCS20T/H-Hr and KCS22T/H-Hr) were obtained. From strains with heterogeneous phenotypes, we selected HCR subpopulations (KCS20T/H-Hr/H and KCS22T/H-Hr/H) by patch plating. Thus, the degree of antibiotic resistance to carbapenem was determined based on the final characteristics of the strains. That is, a case ending with H indicates high resistance and a case ending with L indicates low resistance. Hr denotes the heterogeneous phenotype.

### Antibiotic susceptibility testing

Minimal inhibitory concentrations (MICs) of imipenem, meropenem, ciprofloxacin, tetracycline, amikacin, colistin, azidothymidine, and chloramphenicol were measured for wild-type *K. pneumoniae* isolates (KCS20 and KCS22) and their transformants of pOXA-232 and subsequently obtained strains. The broth microdilution method, following the Clinical and Laboratory Standards Institute (CLSI) guidelines [22], was used for the assay with the reference strains *Escherichia coli* ATCC 25922 and *Pseudomonas aeruginosa* ATCC 27853.

### Time-killing assay

The assay followed the CLSI guidelines [23]. Briefly, overnight bacterial cultures were diluted in a 1:100 ratio in 10 mL of Mueller–Hinton (MH) II broth containing imipenem at a concentration of 8 mg/L. The mixtures were then incubated with constant shaking at 220 rpm for 24 h at 37 °C. Viable cell counts were determined at 0, 3, 6, 9, and 24 h using the spot test method, and the results were expressed as CFU/mL. The experiment was repeated three times.

### Patch plating

Within the transformants with pOXA-232 (KCS20T and KCS22T), the HCR (KCS20T/H and KCS22T/H) and LCR (KCS22T/L and KCS22T/L) populations were identified and isolated using patch patterning. After overnight growth in liquid LB media at 37 °C with constant shaking at 220 rpm, each mixed transformant was spread on non-selective blood agar plates. From each plate, 25 colonies were randomly selected and patched onto new agar plates containing high (32 mg/L) and low (1 mg/L) meropenem concentrations. After overnight incubation at 37 °C, strains that grew on both high and low meropenem concentration agar were classified as having a high carbapenem-resistance (HCR) phenotype. Strains that only grew on low meropenem concentration agar were classified as low carbapenem-resistance (LCR) phenotype. Additionally, strains that grew well on low meropenem concentration agar but formed small spots on high meropenem concentration agar were categorized as heteroresistant (H-Hr). All HCR, LCR and H-Hr were further tested using the antibiotic susceptibility testing and/ or PAP assay to confirm their phenotype.

### Population analysis profiling (PAP)

To identify carbapenem heteroresistance in the strains, a population analysis profiling (PAP) assay was performed as previously mentioned [24] with minor modifications. Overnight cultures were diluted tenfold in phosphate-buffered saline (PBS), and then spread onto MH II agar plates with a two-fold gradient of meropenem concentrations: 0.015 to 8 mg/L for wild types and 2 to 64 mg/L for KCS20T/H-Hr and KCS22T/H-Hr. After incubation for 24 h at 37 °C, CFUs were calculated. Heteroresistance was defined as the presence of resistant subpopulations with MICs at frequencies of 10⁻⁷ to 10⁻⁶.

### Bacterial growth curves

The growth of the wild types and their subsequently obtained transformants was measured as follows. Overnight LB cultures were adjusted to a McFarland standard of 0.5, diluted in a ratio of 1:100 in fresh LB broth, and incubated at 37 °C with shaking at 220 rpm every 24 h for every 2 h. Optical density at 600 nm (OD600) was measured at each time point. Growth curves were analyzed using Student’s t-test and experiments were performed independently three times.

### Competition assay

An in vitro competition assay was performed for overnight cultures of the isogenic transformants, KCS20T/H vs. KCS20T/L and KCS22T/H vs. KCS22T/L. McFarland standard was adjusted to a 0.5 McFarland standard, mixed in a ratio of 1:1 in 10 mL LB broth, and incubated at 37 °C with shaking at 220 rpm for 24 h. The survival rate was assessed by serial tenfold dilutions and spot testing on LB agar plates with meropenem concentrations of 32 and 2 mg/L. The competition index (CI) was calculated as described by Lee et al. [21].

### Phenotype stability assay

The stability of the carbapenem-resistant phenotypes was assessed for 20 days by subculturing the transformants in non-selective liquid media at 37 °C with shaking at 220 rpm. Daily liquid cultures were serially diluted in PBS and spot-tested on Luria–Bertani (LB) agar at high (32 mg/L) and low (1 mg/L) meropenem concentrations. After overnight incubation at 37 °C, CFUs were counted, and the survival ratio was calculated for each phenotype. PCR (primers in Supplementary Table S1) and antimicrobial susceptibility assays confirmed the presence of pOXA-232 and changes in carbapenem resistance.

### Serum bactericidal assay

Human serum resistance assays were performed on wild-type strains and isogenic transformants obtained from them were KCS20T/H, KCS20T/L, KCS22T/H, and KCS22T/L, as previously described [21] with minor modifications. Mid-log phase cultures were treated with normal human serum (NHS) or heat-inactivated human serum (HIS). After 3 h of incubation at 37 °C with shaking at 220 rpm, cultures were washed with PBS, serially diluted, and spot-tested on LB agar. The survival rate was calculated by dividing the CFUs after NHS treatment by the CFUs after HIS treatment. Assays were performed in triplicate for all strains.

### *Galleria mellonella* larvae infection

To compare the virulence of the isogenic transformants, a *G. mellonella* larval infection assay was performed via intra-hemocoelic injection following Kim et al. [25] with minor modifications. We used *G. mellonella* larval infection model because they are inexpensive, but have better ethical acceptability and complete immune system that parallels human innate immunity. KCS20T/H and KCS20T/L were injected at a concentration of 1 × 10^6^ CFU/mL, whereas KCS22T/H and KCS22T/L were injected at a concentration of 1 × 10^5^ CFU/mL. The number of deaths was recorded daily for four days. The injected bacterial concentration and the number of days required to conduct the assay were experimentally determined.

### Carbapenemase, *ompK36*, *ompK35*, and virulence gene expression

Among the transformants of KCS20 and KCS22, quantitative real-time PCR (qRT-PCR) was used to compare mRNA expression levels of *bla*_OXA-232_, virulence genes (*clbA, clbB, ybtS, fyuA, entB, irp2, fimH*, and *traT*), stress response transcription factors (*ramA, soxS, marA, rpoS*, and *clpB*), and porins (*ompK35* and *ompK36*) (Supplementary Table S2). RNA was extracted from mid-log cultures using the RNeasy Mini Kit (Qiagen) and qRT-PCR was performed using the QuantStudio 6 Flex system (Applied Biosystems) with TB Green Premix Ex Taq (TaKaRa). The primers are listed in Supplementary Table S1. The mRNA expression levels were normalized to *rpoB* using the CT method and all assays were performed in triplicate.

### Whole genome sequencing

Whole genome sequences were determined for each HCR and LCR pair as well as for KCS20T/H-Hr and KCS22T/H-Hr. Genomic DNA was extracted using the G-spin^™^ Total DNA Extraction Kit and quantified with NanoDrop. Approximately 100 ng DNA from each sample was used for library preparation. MinION™ sequencing was conducted using the R9.4 flow cells (Oxford Nanopore Technologies) following the manufacturer's instructions, with data acquisition using the MinKNOW software ver. 1.11.5. Short-read sequencing was performed on the same DNA using an Illumina NovaSeq 6000 system (Macrogen, Seoul, South Korea).

### Statistical analyses

Statistical analyses were conducted using Student's *t*-test in Prism version 8.3.0 (GraphPad Software, San Diego, CA, USA). Significance levels were set at *p* < 0.05 (*), *p* < 0.01 (**), or *p* < 0.001 (***).

## Results

### Identification and isolation of two subpopulations

**Fig. 2:**
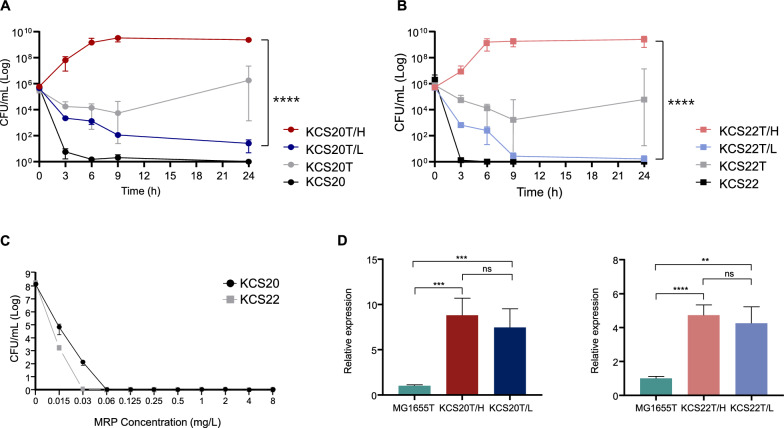
(**A** and **B**) The results of time-killing assays using 8 mg/L imipenem with susceptible wild-types (KCS20 and KCS22), transformants before separation of subpopulation (KCS20T and KCS22T), their subpopulations according to carbapenem resistance levels (KCS20T/H, KCS20T/L, KCS22T/H, and KCS22T/L). (**C**) PAP results against meropenem of wild-types KCS20 and KCS22. Results indicate that both wild-types are initially homogenous in terms of carbapenem susceptibility. ****, *p* < 0.0001. (**D**) The *bla*_OXA-232_ expression of subpopulations of transformants. *E. coli* MG1655 with pOXA-232 were also included. No significant difference in *bla*_OXA-232_ expression was observed between the subpopulations of transformants. **, *p* < 0.01; ***, *P* < 0.001; ****, *p* < 0.0001; ns, not significant

To investigate the effect of OXA-232 on carbapenem resistance according to the genotype, we introduced a plasmid bearing *bla*_OXA-232_ into total of 41 non carbapenem resistant *K. pneumoniae* isolates. We investigated the effect of the *bla*_OXA-232_-bearing plasmid because the introduction of it led to the identificaiton of a heterogeneous transformant populations. Other plasmids with carbapenease genes, for example *bla*_NDM-1_, *bla*_KPC-2_, did not cause the phenomenon when introduced into *K. pneumoniae*. Time-kill analysis of the KCS20T and KCS22T transformants of KCS20 and KCS22, respectively showed high deviations beginning with the 3 h time period, throughout the experiment, which was carried out in triplicate. (Fig. 2A and B). Therefore, we suspected the presence of subpopulations with different levels of carbapenem resistance.

Single colonies of KCS20T and KCS22T were patch-plated on low (2 mg/L) and high (32 mg/L) carbapenem-concentration agar plates, and the transformants harbored heterogeneous subpopulations that exhibited two significantly different carbapenem resistance profiles. Multi-locus sequence typing (MLST) confirmed that the two subpopulations belonged to the identical sequence type ST11, and PCR and Sanger sequencing confirmed the presence of pOXA-232 in both subpopulations. In addition, PAP analysis of the wild types, KCS20, and KCS22, demonstrated no heteroresistance phenotype against meropenem (Fig. 2C), indicating that heterogeneity of carbapenem resistance did not exist in the wild types prior to introduction of the plasmid.

In further time-killing assays with 8 mg/L imipenem, the HCR subpopulations (KCS20T/H and KCS22T/H) showed increased survival from the beginning of culture (Fig. 2A and B). However, survival of the LCR populations (KCS20T/L and KCS22T/L) decreased and the rates were slow compared to the susceptible wild types.

### Variation in carbapenem resistance between subpopulations of transformants and expression of *bla*_OXA-232_

The two subpopulations of transformants showed more than a 16-fold difference in carbapenem MICs. Although the HCR subpopulations KCS20T/H and KCS22T/H exhibited carbapenem MIC above 64 mg/L, the LCR subpopulations KCS20T/L and KCS22T/L showed carbapenem MIC of 4 mg/L (Table 1). The transformants showed no increase in MIC with respect to the other antibiotics, and no differences in antibiotic susceptibility profiles, except for carbapenems, were identified between the two subpopulations of each pair.

The *bla*_OXA-232_ expression levels between the subpopulations of transformants in each pair were assessed and normalized to that of the *E. coli* reference strain MG1655 transconjugated with pOXA-232. Both subpopulations with pOXA-232 in both pairs exhibited a significant increase in *bla*_OXA-232_ expression compared to that in the reference strain, but there was no significant difference between the subpopulations of each pair (Fig. 2D).

### Growth, fitness, and phenotype stability

**Fig. 3:**
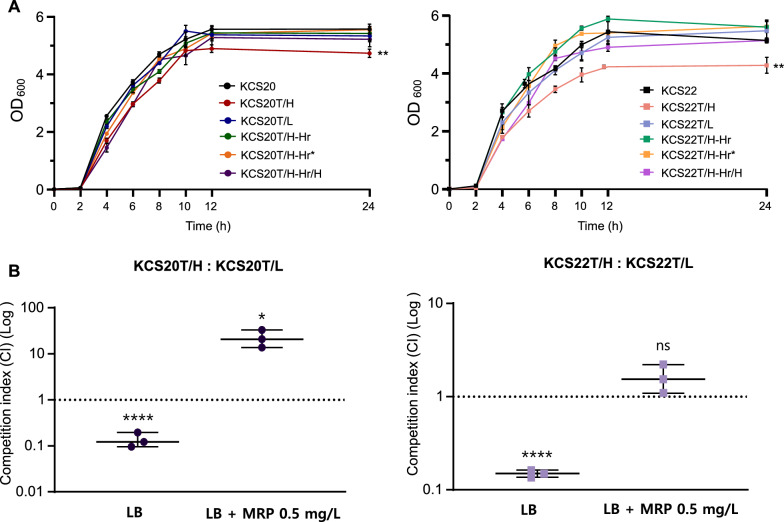
(**A**) Growth curve analysis of KCS20, KCS22, and their subsequent mutants. (**B**) Results of competition assay for HCR and LCR subpopulations of each pair. A value greater than 1 means that HCR subpopulations are more competitive, while a value less than 1 means that HCR subpopulations are less competitive. **, *p* < 0.01

Growth curve analysis showed that LCR subpopulations had similar growth rates to their wild-type counterparts, but HCR subpopulations (KCS20T/H and KCS22T/H) demonstrated significantly lower growth than the wild-type and LCR subpopulations (Fig. 3A). However, HCR subpopulations obtained from resilient heterogeneous strains in media without antibiotics, KCS20T/H-Hr/H and KCS22T/H-Hr/H, recovered the growth rates of the wild types.

The in vitro competitiveness was assessed between the isogenic transformant pairs KCS20T/H vs. KCS20T/L and KCS22T/H vs. KCS22T/L (Fig. 3B). Without meropenem, the LCR subpopulations outcompeted the HCR subpopulations in both pairs. However, HCR subpopulations significantly outcompeted LCR subpopulations at low meropenem (0.5 mg/L).

**Fig. 4:**
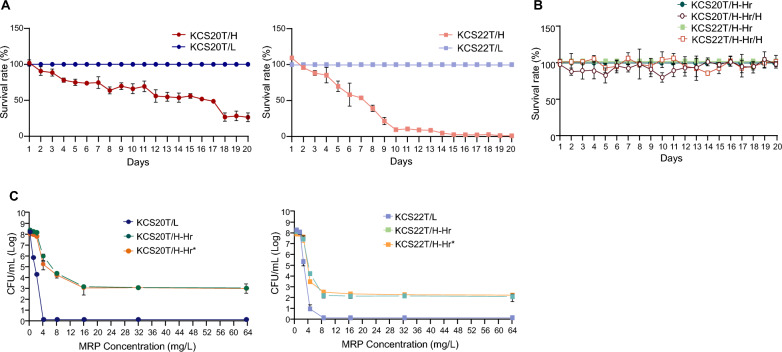
(**A**) Results of phenotype stability assay for the HCR and LCR subpopulations in each transformant. In both pairs, individual subculturing of phenotype HCR subpopulations (KCS20T/H and KCS22T/H) lead to a decrease in the HCR phenotype, and majority of the population consisted of LCR phenotype (**B**) Results of phenotype stability assay for heterogeneous strains obtained from HCR subpopulations of transformants. In addition, HCR subpopulations isolated from the heterogeneous strains were also subjected to phenotype stability assay. (**C**) Population analysis profiling results against meropenem

Phenotypic stability analysis showed that HCR was unstable. While KCS20T/L and KCS22T/L cells preserved their carbapenem-resistant phenotypes, KCS20T/H and KCS22T/H showed decreased survival rates during the 20-day subculture in antibiotic-free media (Fig. 4A). In addition, the LCR phenotype emerged during subculturing of the HCR subpopulations. Approximately half of the total population by day 7 for both HCR subpopulations was composed of subpopulations with LCR resistance phenotype. Strains with heterogeneous phenotypes recovered after 20-day subculturing of KCS20T/H and KCS22T/H were isolated and termed KCS20T/H-Hr and KCS22T/H-Hr, respectively.

KCS20T/H-Hr and KCS22T/H-Hr were found to be stable through the phenotypic stability analysis (Fig. 4B). We performed PAP analysis for KCS20T/H-Hr and KCS22T/H-Hr cells, in addition to the initial LCR subpopulations, KCS20T/L and KCS22T/L. These results revealed that KCS20T/H-Hr and KCS22T/H-Hr were heterogeneous (Fig. 4C). That is, the HCR subpopulations recovered their heterogeneity during subculture rather than converting to LCR. The proportion of HCR subpopulations was approximately 10^–6^. The HCR subpopulations (KCS20T/H-Hr/H and KCS22T/H-Hr/H) (carbapenem MICs, 64 mg/L) were differentiated from the secondary heterogeneous strains by patch plating. The HCR subpopulations showed no decrease in growth rate (Fig. 3A) and their resistant phenotype was stable (Fig. 4B).

When the secondary heterogeneous strains were subcultured in media without antibiotics, the resulting strains (KCS20T/H-Hr* and KCS22T/H-Hr*) preserved their growth rate and heterogeneous phenotype of carbapenem resistance (Fig. 3A and 4C).

### Variations in virulence between subpopulations of transformants

**Fig. 5:**
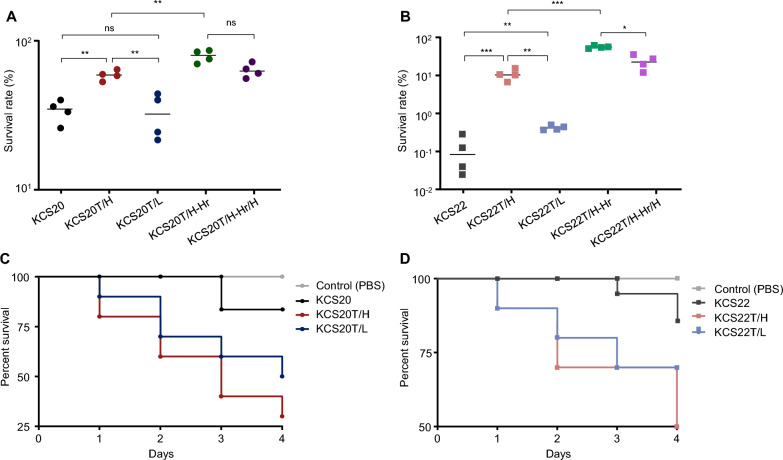
(**A** and **B**) Results of human serum survival assay. HCR subpopulations and heterogeneous strains showed higher survival against human serum for both pairs in comparison to wild-types and LCR subpopulations. (**C** and **D**) Results of *G. mellonella* larvae infection assay. No statistical differences between subpopulations were observed. *, *p* < 0.05; **, *p* < 0.01; ***, *p* < 0.001

In in vitro human serum assay, HCR subpopulations exhibited higher survival rates than LCR subpopulations in both transformant pairs (Fig. 5A and B). The HCR subpopulations also survived significantly better than their corresponding wild-type counterparts.

In the in vivo* G. mellonella* infection assay, both the HCR and LCR subpopulations showed higher virulence than their corresponding wild types (Fig. 5C and D). However, no statistically significant differences were observed between the HCR and LCR subpopulations in each pair.

**Fig. 6:**
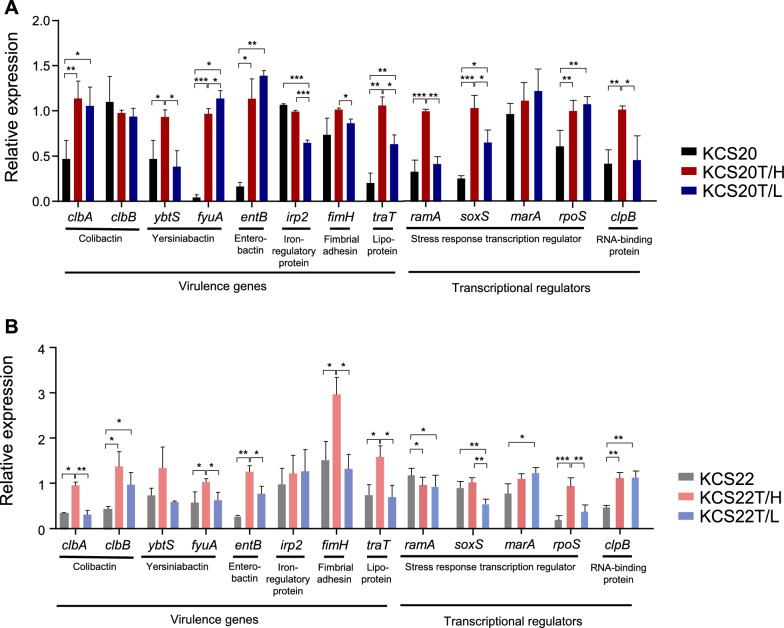
(**A** and **B**) Expression of virulence genes and transcription regulators, predominantly involved in human serum survival, in wild-types, and subpopulations of transformants. *, *p* < 0.05; **, *p* < 0.01; ***, *p* < 0.001

The expression of virulence-associated and transcriptional regulatory genes was measured. Although many genes showed incongruent expression patterns between strains, *fimH*, *traT*, and *soxS* were significantly upregulated in both HCR populations compared to the LCR subpopulations (Fig. 6).

### Genomic comparison

**Fig. 7:**
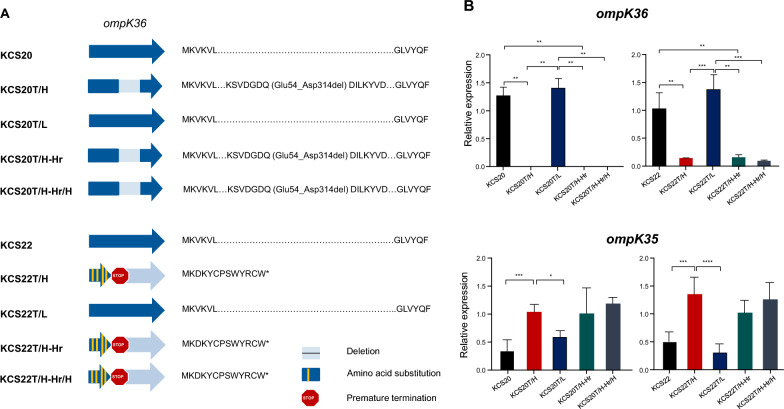
(**A**) Comparison of *ompK36* sequences in two pairs. Deletion region, amino acid substitution, and premature termination were represented schematically, and predicted amino acid sequences were also indicated. Dots indicate abbreviated amino acids, and asterisk (*) indicates premature termination. (**B**) Comparison of *ompK36* and *ompK35* expression in each pair. Significant difference in *ompK36* and *ompK35* expression was observed between the HCR and LCR transformants of each pair. *, *p* < 0.05; **, *p* < 0.01; ***, *p* < 0.001; ****, *p* < 0.0001

Several single-nucleotide polymorphisms (SNPs) were identified using whole-genome sequencing. Among these, only one disruption in *ompK36* was observed. Variations in *ompK36* expression are associated with carbapenem resistance. Different alterations in *ompK36* were identified in the two HCR subpopulations, KCS20T/H and KCS22T/H (Fig. 7A). In KCS20T/H, a 259 bp deletion (Glu54_Asp314del) was identified in *ompK36*, which led to the production of dysfunctional OmpK36. An amino acid substitution that causes a frameshift mutation leading to premature termination was found in *ompK36* of KCS22T/H. No mutations were found in *ompK36* in the LCR subpopulations, KCS20T/L and KCS22T/L (Fig. 7A). These results were further confirmed in mRNA expression analysis of *ompK36*, in which *ompK36* was either not expressed or expressed at very low levels in KCS20T/H and KCS22T/H cells (Fig. 7B).

Genetic alterations in *ompK36* were preserved in the subsequently obtained strains, KCS20T/H-Hr, KCS20T/H-Hr/H, KCS22T/H-Hr, and KCS22T/H-Hr/H (Fig. 7A). The mRNA expression pattern reflected the genetic alterations in *ompK36* (Fig. 7B). In addition, we confirmed that *ompK35* and *ompK36* functioned reciprocally with one another (Fig. 7B); that is, higher *ompK35* expression was observed in strains with no or lower *ompK36* expression.

### Genetic alterations in heterogeneous strains

In addition to *ompK36,* other genetic alterations were identified in KCS20T/H-Hr and KCS22T/H-Hr. In both heterogeneous strains, a deletion of approximately 9,000 bp was found, which included *yedR, yedJ, dcm*, *vsr, yedA, yedI, dgcQ*, *dsrB*, *rcsA*, and two hypothetical genes (Supplementary Figure S1). The KCS20T/H-Hr strain featured a 702 bp sequence insertion, which included an Is1-like element, IsKpn14 family transposase, and a partial Is1 repressor protein (InsA), within the 9,000 bp deletion region. This insertion was absent in the KCS22T/H-Hr strain. Initially, the insertion sequence was located between positions 4,499,134 and 4,499,835 in the genomes of both the wild-type and KCS20T/H strains. The deletion was maintained in the subsequently isolated stable HCR subpopulations KCS20T/H-Hr/H and KCS22T/H-Hr/H.

## Discussion

Carbapenem resistance is a significant concern, especially in *K. pneumoniae*, due to its association with high morbidity and mortality [3]. *K. pneumoniae* employs various mechanisms to resist treatment, including overexpression of efflux pumps, reduced permeability, and carbapenemase production [26]. Additionally, population heterogeneity, such as heteroresistance, accelerates the evolution of resistance [27].

In this study, we examined the heterogeneity of carbapenem resistance and its associated growth rate, fitness, and virulence in isogenic transformants by introducing a carbapenemase gene (*bla*_OXA-232_)-harboring plasmid into a carbapenem-susceptible *K. pneumoniae* strain. Two transformant pairs (KCS20T and KCS22T) with the HCR and LCR subpopulations were identified. While the LCR population showed a carbapenem MIC of 4 mg/L, the HCR subpopulation exhibited a carbapenem MIC > 64 mg/L. HCR subpopulations were less competitive than isogenic LCR subpopulations in a carbapenem-free environment. The lower growth rate of the HCR subpopulations support their lower competitiveness. In addition, the HCR phenotype may be unstable, as prolonged sub-culturing without antibiotics leads to a decline in the HCR phenotype, resulting in the LCR phenotype.

The qRT-PCR analysis of the transformants revealed that both the HCR and LCR strains expressed similar levels of *bla*_OXA-232_ in a carbapenem-free environment, indicating that the difference in carbapenem MIC was not due to the differential expression of *bla*_OXA-232_ but due to other mechanisms. The HCR phenotype is likely associated with dysfunction of *ompK36*. OmpK36 is the main porin that allows carbapenems to enter the cells [28]. Many studies have shown that complete loss or low expression of *ompK36* leads to carbapenem resistance in *K. pneumoniae* [29, 30]. Restoring *ompK36* function in defective isolates has been shown to reduce carbapenem resistance [31], while overexpression of *ompK36* has been associated with increased carbapenem susceptibility [32]. Although OXA-232 contributes to decreased carbapenem susceptibility to low-level resistance, defective OmpK36 may have amplified carbapenem resistance to a high level.

HCR subpopulations also exhibited higher expression of virulence genes, such as *fimH* (fimbrial adhesin gene), *traT* (lipoprotein gene), and the transcription regulator *soxS* (stress response gene) than LCR subpopulations. These genes are known to affect the virulence of Enterobacterales, including human serum resistance [33, 34]. Studies have also shown that the loss of *ompK36* leads to high human serum resistance but no difference in the macrophage internalization rate was observed [35, 36]. Thus, the loss of *ompK36* functionality may be responsible for the increased virulence phenotype, including resistance to human serum in HCR subpopulations, by affecting other genes.

Other studies have demonstrated similar phenomena with carbapenem-susceptible isolates producing weak carbapenemases *bla*_KPC-2_ and *bla*_OXA-48_ with the spontaneous formation of carbapenem-resistant subpopulations harboring defects in *ompK36* [37, 38]. Although the loss of *ompK36* functionality is reversible in antibiotic-free media in some cases, it is permanent in others, resulting in high level of resistance [39, 40]. Our results, in accordance with those of previous studies, may indicate that heterogeneity due to mutations in *ompK36* is a more common phenomenon than previously thought.

Moreover, the LCR strain obtained by subculturing HCR subpopulations was revealed to have a heterogeneous phenotype. This differs from classical heteroresistance in that it is not carbapenem susceptible in in vitro antimicrobial susceptibility testing. Instead, the main population was LCR with a subpopulation of HCR. In other words, the heterogeneous phenotypes were resilient.

A stable HCR phenotype was induced in the heterogeneous strain. Stable HCR strains displayed a higher growth rate than unstable strains. Deficient *ompK36* was preserved, but an additional compensatory mutation was found. The 9,000 bp-deleted region found in the stable HCR strains included *dcm* (DNA cytosine methyltransferase), vsr (strand- and sequence-specific DNA mismatch endonuclease), *dsrB* (dissimilatory-type sulfite reductase subunit beta), and *rcsA* (regulation of serotype-specific K antigen expression) (Supplementary Figure S1). They are also involved in DNA methylation, mismatch repair, sulphite reduction, and capsular antigen expression [41–43]. Among the genes, *dcm* is a cytosine-specific methyltransferase that specifically methylates cytosines in DNA, recognizing the double-stranded sequence 5'- CCWGG-3', methylates C-2 on both strands. Thus, it represses the transcription of the affected gene(s) [41]. The absence of *dcm* may have demethylated additional genes that could compensate for the reduced fitness, potentially contributing to the emergence of stable HCR subpopulations from the initially unstable HCR. This suggests a ‘bet-hedging’ strategy, in which heterogeneous phenotypes enhance survival under changing conditions. The fast-growing LCR phenotype is favored in antibiotic-free environments, whereas the HCR subpopulation thrives under carbapenem stress, ultimately stabilizing the HCR phenotype, which is highly fit and highly resistant to carbapenems.

OXA-232 is also associated with low carbapenem resistance [44]. It sometimes causes high levels of carbapenem resistance when combined with extended-spectrum *β*-lactamases [45]. The loss of function of outer membrane proteins, as shown in this study, may also be responsible for the high level of carbapenem resistance coupled with high virulence [46].

## Conclusions

This study underscores the importance of recognizing hidden heterogeneity within clinical pathogenic populations, even though heterogeneous strains have been identified in factitious transformants. Phenotypically silent under normal conditions, heterogeneity can lead to significant differences in antibiotic resistance and virulence under certain circumstances, such as the introduction of plasmids harboring the carbapenemase gene. Although our findings were limited to only two *K. pneumoniae* isolates, undetected heterogeneity could lead to stable high antibiotic resistance, which is responsible for treatment failure. Understanding these mechanisms is vital for addressing carbapenem monotherapy failures and infection relapses, as well as for developing strategies to mitigate the impact of antibiotic resistance heterogeneity in clinical settings.

## Supplementary Information

Supplementary material 1. Figure S1. Compensatory mutation region found in both KCS20T/H-Hr and KCS22T/H-Hr.

Supplementary material 2.

## Data Availability

All materials are available upon request to the corresponding author.

## Abbreviations

CFU: Colony-forming unit
CI: Competition index
CLSI: Clinical and Laboratory Standards Institute
HIS: Heat-inactivated human serum
NHS: Normal human serum
KPC: *Klebsiella pneumoniae* Carbapenemase
LB: Luria–Bertani
MIC: Minimum inhibitory concentration
NDM: New Delhi metallo-β-lactamase
OXA: Oxacillinase
PBS: Phosphate-buffered saline
