# Supplementary figures and images for: Emergence of population heterogeneity in Klebsiella pneumoniae with a blaOXA-232-harboring plasmid: carbapenem resistance, virulence, and fitness
Source: J Biomed Sci. 2025 Feb 15;32:22. doi: 10.1186/s12929-024-01108-4 (PMC11829361; doi:10.1186/s12929-024-01108-4)

Suppl. Fig. 1

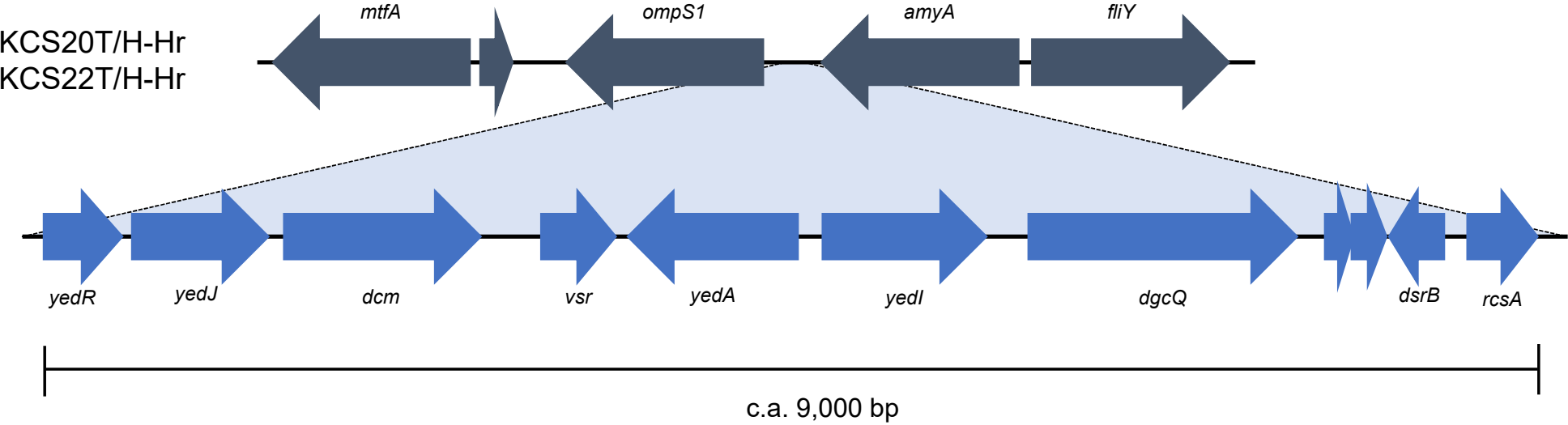

Supplement: Supplementary file 1 — Supplementary material 1. Figure S1. Compensatory mutation region found in both KCS20T/H-Hr and KCS22T/H-Hr. [file 12929_2024_1108_MOESM1_ESM.pdf]
