# Supplementary material for: Emergence of population heterogeneity in Klebsiella pneumoniae with a blaOXA-232-harboring plasmid: carbapenem resistance, virulence, and fitness
Source: J Biomed Sci. 2025 Feb 15;32:22. doi: 10.1186/s12929-024-01108-4 (PMC11829361; doi:10.1186/s12929-024-01108-4)
Supplement: Supplementary file 2 — Supplementary material 2. [file 12929_2024_1108_MOESM2_ESM.pdf]

**Supplementary Table S1.** Description of strains and plasmid used in this study. SMC stands for Samsung Medical Centre, Seoul

| Bacteria/ Plasmid | Description |
|---|---|
| **Wild types** | |
| KCS20 | Carbapenem susceptible clinically isolated *K. pneumioniae* from Samsung Medical Center (Seoul, South Korea) |
| KCS22 | Carbapenem susceptible clinically isolated *K. pneumioniae* from Samsung Medical Center (Seoul, South Korea) |
| **Isogenic transformants** | |
| KCS20T/H | pOXA-232 transformant of high carbapenem resistance and serum resistance phenotype created from KCS20, produces *bla*$_{OXA-232}$. |
| KCS20T/L | pOXA-232 transformant of low carbapenem resistance and serum resistance phenotype created from KCS20, produces *bla*$_{OXA-232}$. |
| KCS20T/H-H | pOXA-232 transformant with heterogeneous phenotype separated after 20 days of subculturing KCS20T/H, produces *bla*$_{OXA-232}$. |
| KCS20T/H-Hr/H | pOXA-232 transformant with high carbapenem resistance phenotype separated after 20 days of subculturing KCS20T/H-Hr, produces *bla*$_{OXA-232}$. |
| KCS22T/H | pOXA-232 transformant of high carbapenem resistance and serum resistance phenotype created from KCS22, produces *bla*$_{OXA-232}$. |
| KCS22T/L | pOXA-232 transformant of low carbapenem resistance and serum resistance phenotype created from KCS22, produces *bla*$_{OXA-232}$. |
| KCS22T/H-Hr | pOXA-232 transformant with carbapenem heteroresistant phenotype separated after 20 days of subculturing KCS22T/H, produces *bla*$_{OXA-232}$. |
| KCS22T/H-Hr/H | pOXA-232 transformant with high carbapenem resistant phenotype separated after 20 days of subculturing KCS22T/H-Hr, produces *bla*$_{OXA-232}$. |
| **Plasmid** | |
| pOXA-232 | Plasmid 6,141 bp in size. Harbors replicase gene, *bla*$_{OXA-232}$, hypothetical gene, a mobile gene cassette (MOB module), and one noncoding partial gene EreA, erythromycin resistance, and coding partial vbhA gene plasmid. Isolated from clinical isolate *K. pneumoniae* M5 (KM5). |

**Supplementary Table S2.** Primers used in this study.

| Primers | | Sequences (5' to 3' Direction) | References |
|---|---|---|---|
| **pOXA-232 detection primers** | pOXA-232_ORI-F | CTTGGGCGAAACTGTACCCT | This study |
| | pOXA-232_ORI-R | AGGCTTGTTTCGATCACGGA | |
| | $bla_{OXA-232}$-FW | GGCTGTGTTTTTGGTGGCAT | This study |
| | $bla_{OXA-232}$-RV | CCCAAAGCGCCTCTGTAGAA | |
| ***ompK* detection primers** | *ompK35*_FW | CCTGGCGGAGTTCTGGAT | This study |
| | *ompK35*_RV | TCGGTCAGTTCCTGCTCCC | |
| | *ompK36*_FW | TCCATTAATCGAGGCTCCT | This study |
| | *ompK36*_RV | TAGTGCGTATTTCCCTGACC | |
| **Virulence gene detection Primers** | *rmpA*-F | CATAAGAGTATTGGTTGACAG | |
| | *rmpA*-R | CTTGCATGAGCCATCTTTCA | Compain *et al.*, 2014 |
| | *kfu*-F | GGCCTTTGTCCAGAGCTACG | |
| | *kfu*-R | GGGTCTGGCGCAGAGTATGC | |
| | *mrkD*-F | AAGCTATCGCTGTACTTCCGGCA | |
| | *mrkD*-R | GGCGTTGGCGCTCAGATAGG | |
| | *ybtS*-F | GACGGAAACAGCACGGTAAA | |
| | *ybtS*-R | GAGCATAATAAGGCGAAAGA | |
| | *iutA*-F | GGGAAAGGCTTCTCTGCCAT | |
| | *iutA*-R | TTATTCGCCACCACGCTCTT | |
| | *allS*-F | CATTACGCACCTTTGTCAGC | |
| | *allS*-R | GAATGTGTCGGCGATCAGCTT | |
| | *clbA*-F | ATGAGGATTGATATATTAATTGGAC | Khaertynov *et al.* 2018 |
| | *clbA*-R | ATTCTGCCCATTTGACGAATG | |
| | *clbB*-F | GATTTGGATACTGGCGATAACCG | |
| | *clbB*-R | CCATTTCCCGTTTGAGCACAC | |
| | *iro*-F | GTCCGGCGGTAACTTCAGCC | Lee *et al.*, 2016 |
| | *iro*-R | TCAGAATGAAACTACCGCCC | This study |
| | *fimH*-F | GAAAAAAATAATCCCCCTGTTCAC | This study |
| | *fimH*-R | GTAACCTGGCCTGTGGTC | This study |
| | *ycfM*-F | ATCAGCAGTCGGGTCAGC | This study |
| | *ycfM*-R | CTTCTCCAGCATTCAGCG | This study |
| | *entB*-F | ATTTCCTCAACTTCTGGGGC | This study |
| | *entB*-R | AGCATCGGTGGCGGTGGTCA | This study |
| | *irp2*_F | GCTACAATGGGACAGCAACGAC | This study |
| | *irp2*_R | GCAGAGCGATACGGAAAATGC | This study |
| | *traT*_F | GGTGTGGTGCGATGAGCACAG | This study |
| | *traT*_R | CACGGTTCAGCCATCCCTGAG | This study |
| **qRT-PCR primers** | qrt_*rpoB*_KPN _F | CGCGTATGTCCFATCGAAA | This study |
| | qrt_*rpoB*_KPN _R | GCGTCTCAAGGAAGCCATATTC | This study |
| | qrt_$bla_{OXA-232}$_F | CGCATCGTGAAACAAGCCAT | This study |
| | qrt_$bla_{OXA-232}$_R | ATGGCTTGTTTCACGATGCG | This study |
| | qRT_*fimH*_FW | GACCAACAACTACAATAGCGAC | This study |
| | qRT_*fimH*_RV | ATTGGTGAAGATCGCGTTGG | This study |
| | qRT_*entB*_FW | ATATCCCGGCGAACAAGGTC | This study |
| | qRT_*entB*_RV | CGGCGATATTAGCCACCACT | This study |
| | qRT_ *ycfM*_FW | GCCGTCTGTGCCAACGATTC | This study |
| | qRT_ *ycfM*_RV | ATCGCGCCGTTCCAGTCATA | This study |
| | qRT_*hfq*_FW | GAAGAACACGGTCAGCCAGA | This study |
| | qRT_*hfq*_RV | ATAGTTACTGGAACCGCCGC | This study |
| | qRT_*soxS*_FW | AGTCGCCAGAAAGTCAGGAT | This study |
| | qRT_*soxS*_RV | GCATCACGGTACGGAACAT | This study |

| | | |
|---|---|---|
| qRT_*marA*_FW | ATGTACTGGCCGAGGGAAT | This study |
| qRT_*marA*_RV | AGCGCTCCGGTTACTCTAAG | This study |
| qRT_*rpoS*_FW | CAGCTCTTCCTCAGCCAGAT | This study |
| qRT_*rpoS*_RV | ATGATTTAAATGAAGACGCGGA | This study |
| qrt_*clbB*_FW | TTAGTCGAGGGCGGGATACA | This study |
| qRT_*clbB*_RV | GATTTGCCCCAGCAACTGTG | This study |
| qRT_*traT*_FW | TATGAGGGCGCTGTGACTGG | This study |
| qRT_*traT*_RV | AGCAGCCATACCAACCAGAC | This study |
| qRT_*fyuA*_FW | TCCCTCAGCTTTCCACCAAC | This study |
| qRT_*fyuA*_RV | GATGTTGATGATCCCGCCCT | This study |
| qRT_*ramA*_FW | ATCGTCGAGTGGATTGATGA | This study |
| qRT_*ramA*_RV | AGATGCCATTTCGAATACCC | This study |
| qRT_*clpB*_FW | CAGCACTTCGGCGATTTCAG | This study |
| qRT_*clpB*_RV | TCCGGAACTGGAGAAACAGC | This study |
| qRT_*hfq*_FW | GAAGAACACGGTCAGCCAGA | This study |
| qRT_*hfq*_RV | ATAGTTACTGGAACCGCCGC | This study |
| qRT_*soxS*_FW | AGTCGCCAGAAAGTCAGGAT | This study |
| qRT_*soxS*_RV | GCATCACGGTACGGAACAT | This study |
| qRT_*marA*_FW | ATGTACTGGCCGAGGGAAT | This study |
| qRT_*marA*_RV | AGCGCTCCGGTTACTCTAAG | This study |
| qRT_*rpoS*_FW | CAGCTCTTCCTCAGCCAGAT | This study |
| qRT_*rpoS*_RV | ATGATTTAAATGAAGACGCGGA | This study |
| qRT_*ompK35*_FW | AGCGACGATACCACCTATGC | This study |
| qRT_*ompK35*_RV | ACGCGTCCATGTTGTATTCC | This study |
